# 3,9-Dibromo-5,7-dihydro­dibenzo[*c*,*e*]oxepine

**DOI:** 10.1107/S1600536808018175

**Published:** 2008-06-19

**Authors:** Hai-Quan Zhang, Bao-Li, Guang-Di Yang, Yu-Guang Ma

**Affiliations:** a State Key Laboratory of Metastable Materials Science and Technology, Yanshan University, Qinhuangdao 066004, People’s Republic of China; b State Key Laboratory of Supramolecular Structure and Materials, Jilin University, Changchun 130012, People’s Republic of China

## Abstract

The title compound, C_14_H_10_Br_2_O, is a biphenyl derivative containing a –CH_2_—O—CH_2_– bridge in the 2,2′-position. The compound displays a twisted conformation with the two benzene rings making a dihedral angle of 45.02 (5)°, while the central seven-membered ring is in a boat conformation. The mol­ecule lies on a crystallographic twofold axis of symmetry passing through the O atom and bis­ecting the 1,1′ C—C bond.

## Related literature

For a previous synthesis of related biphenyl mol­ecules, see: Mislow & Glass (1961 ▶).
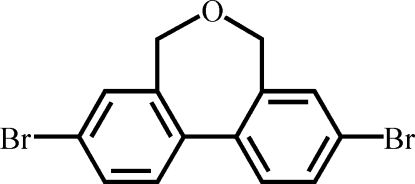


## Experimental

### 

#### Crystal data

C_14_H_10_Br_2_O
*M* *_r_* = 354.04
Orthorhombic, 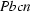

*a* = 16.5965 (3) Å
*b* = 10.2476 (6) Å
*c* = 7.2626 (14) Å
*V* = 1235.2 (2) Å^3^
*Z* = 4
Mo *K*α radiation
μ = 6.54 mm^−1^
*T* = 291 (2) K
0.14 × 0.14 × 0.12 mm

#### Data collection

Rigaku R-AXIS RAPID diffractometer
Absorption correction: multi-scan (*ABSCOR*; Higashi, 1995 ▶) *T*_min_ = 0.457, *T*_max_ = 0.498 (expected range = 0.419–0.456)
2468 measured reflections
1371 independent reflections
896 reflections with *I* > 2σ(*I*)
*R*_int_ = 0.014

#### Refinement

*R*[*F* ^2^ > 2σ(*F* ^2^)] = 0.024
*wR*(*F* ^2^) = 0.042
*S* = 1.05
1371 reflections
78 parameters
H-atom parameters constrained
Δρ_max_ = 0.28 e Å^−3^
Δρ_min_ = −0.46 e Å^−3^

### 

Data collection: *RAPID-AUTO* (Rigaku, 1998 ▶); cell refinement: *RAPID-AUTO*; data reduction: *CrystalStructure* (Rigaku/MSC, 2002 ▶); program(s) used to solve structure: *SHELXS97* (Sheldrick, 2008 ▶); program(s) used to refine structure: *SHELXL97* (Sheldrick, 2008 ▶); molecular graphics: *PLATON* (Spek, 2003 ▶); software used to prepare material for publication: *SHELXL97*.

## Supplementary Material

Crystal structure: contains datablocks global, I. DOI: 10.1107/S1600536808018175/bv2098sup1.cif

Structure factors: contains datablocks I. DOI: 10.1107/S1600536808018175/bv2098Isup2.hkl

Additional supplementary materials:  crystallographic information; 3D view; checkCIF report

## Figures and Tables

**Fig. 1.**
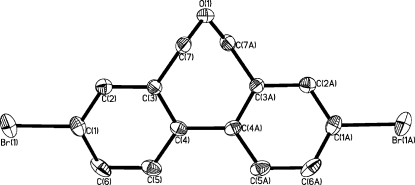
The structure of the title compound, with the atom-labelling Displacement ellipsoids are drawn at the 30% probability level of arbitrary radii.

**Fig. 2.**
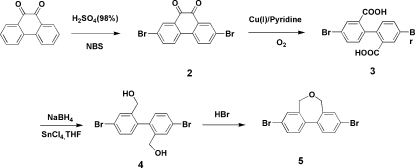
The synthesis route for the preparation of 3,9-dibromo-5,7-dihydro-dibenzo[c,e]oxepine.

## supplementary crystallographic information

### Comment

The dibenzo[c,e]oxepine derivatives were studied due to their optical activity as discussed in a previous article (Mislow & Glass, 1961). Introducing functional groups such as Br on the benzene ring of the dibenzo[c,e]oxepine can expand the range of their applications, such as photoluminescence, electro-luminescence devices and nonlinear optics. Herein we present the crystal structure of the title compound. In orthorhombic (space group Pbcn) crystals of 3,9-dibromo-5,7-dihydro-dibenzo[c,e]oxepine, there are four molecules in the unit cell. The molecule lies on a crystallographic 2-fold axis of symmetry passing through the O and bisecting the C4-C4a bond. The compound exhibits twisted conformation between two phenyl rings with a dihedral angle of 45.02 (5)°, while central 7-member ring is in a boat conformation.

### Experimental

The four-step reaction to prepare 3,9-dibromo-5,7 -dihydro dibenzo [c,e] oxepin is described as follows: (1) 2,7-Dibromo-phenanthrenequinone was obtained by directly brominating phenanthrenequinone in presence N-bromosuccinamide (NBS) in H_2_SO_4_. (2) This was followed by oxidation of 2,7-dibromophenanthrenequinone in the presence of pure oxygen and Cu(I)Cl to give 4,4-dibromodiphenic acid. (3). The reduction of 4,4-dibromodiphenic acid using NaBH_4_ gave 4,4'-dibromo-2,2'-bis-(hydroxymethyl)-biphenyl. (4) The final production was obtained by ring closure of 4,4'-dibromo-2,2'-bis-(hydroxymethyl)-biphenyl in the presence of HBr acid. Single-crystals of X-ray diffraction quality were grown by slow evaporation of a ethanol solution.

### Refinement

C-bound H atoms were geometrically positioned with C—H = 0.97 Å, *U*_iso_(H) = 1.5*U*_eq_(C) for methyl and C—H = 0.93 Å, *U*_iso_(H) = 1.2*U*_eq_(C) for carbon atoms.

### Crystal data

**Table:**

C_14_H_10_Br_2_O	*F*_000_ = 688
*M**_r_* = 354.04	*D*_x_ = 1.904 Mg m^−^^3^
Orthorhombic, *P**b**c**n*	Mo *K*α radiation λ = 0.71073 Å
Hall symbol: -P 2n 2ab	Cell parameters from 10356 reflections
*a* = 16.5965 (3) Å	θ = 2.5–54.9º
*b* = 10.2476 (6) Å	µ = 6.54 mm^−^^1^
*c* = 7.2626 (14) Å	*T* = 291 (2) K
*V* = 1235.2 (2) Å^3^	Block, colorless
*Z* = 4	0.14 × 0.14 × 0.12 mm

### Data collection

**Table:**

Rigaku R-AXIS RAPID diffractometer	1371 independent reflections
Radiation source: fine-focus sealed tube	896 reflections with *I* > 2σ(*I*)
Monochromator: graphite	*R*_int_ = 0.014
*T* = 291(2) K	θ_max_ = 27.5º
ω scans	θ_min_ = 2.3º
Absorption correction: multi-scan(ABSCOR; Higashi, 1995)	*h* = −21→21
*T*_min_ = 0.457, *T*_max_ = 0.498	*k* = −13→12
2468 measured reflections	*l* = −9→9

### Refinement

**Table:**

Refinement on *F*^2^	Secondary atom site location: difference Fourier map
Least-squares matrix: full	Hydrogen site location: inferred from neighbouring sites
*R*[*F*^2^ > 2σ(*F*^2^)] = 0.024	H-atom parameters constrained
*wR*(*F*^2^) = 0.042	*w* = 1/[σ^2^(*F*_o_^2^) + (0.0103*P*)^2^] where *P* = (*F*_o_^2^ + 2*F*_c_^2^)/3
*S* = 1.05	(Δ/σ)_max_ < 0.001
1371 reflections	Δρ_max_ = 0.28 e Å^−^^3^
78 parameters	Δρ_min_ = −0.46 e Å^−^^3^
Primary atom site location: structure-invariant direct methods	Extinction correction: none

### Special details

**Table:**

Geometry. All e.s.d.'s (except the e.s.d. in the dihedral angle between two l.s. planes) are estimated using the full covariance matrix. The cell e.s.d.'s are taken into account individually in the estimation of e.s.d.'s in distances, angles and torsion angles; correlations between e.s.d.'s in cell parameters are only used when they are defined by crystal symmetry. An approximate (isotropic) treatment of cell e.s.d.'s is used for estimating e.s.d.'s involving l.s. planes.
Refinement. Refinement of *F*^2^ against ALL reflections. The weighted *R*-factor *wR* and goodness of fit *S* are based on *F*^2^, conventional *R*-factors *R* are based on *F*, with *F* set to zero for negative *F*^2^. The threshold expression of *F*^2^ > σ(*F*^2^) is used only for calculating *R*-factors(gt) *etc*. and is not relevant to the choice of reflections for refinement. *R*-factors based on *F*^2^ are statistically about twice as large as those based on *F*, and *R*- factors based on ALL data will be even larger.

### Fractional atomic coordinates and isotropic or equivalent isotropic displacement parameters (Å^2^)

**Table:**

	*x*	*y*	*z*	*U*_iso_*/*U*_eq_	
Br1	0.677363 (15)	0.36711 (3)	0.14224 (4)	0.04966 (11)	
O1	1.0000	0.0746 (2)	0.2500	0.0401 (7)	
C1	0.79077 (14)	0.3698 (3)	0.1813 (3)	0.0333 (6)	
C6	0.82561 (17)	0.4814 (3)	0.2538 (3)	0.0392 (7)	
H6A	0.7943	0.5534	0.2846	0.047*	
C3	0.91882 (13)	0.2642 (2)	0.1600 (3)	0.0251 (5)	
C7	0.96934 (14)	0.1504 (2)	0.1017 (3)	0.0342 (6)	
H7A	0.9373	0.0944	0.0228	0.041*	
H7B	1.0143	0.1825	0.0293	0.041*	
C4	0.95561 (13)	0.3761 (2)	0.2353 (3)	0.0273 (5)	
C2	0.83600 (13)	0.2626 (2)	0.1339 (3)	0.0295 (6)	
H2A	0.8112	0.1891	0.0845	0.035*	
C5	0.90852 (16)	0.4830 (3)	0.2793 (3)	0.0361 (7)	
H5A	0.9329	0.5574	0.3270	0.043*	

### Atomic displacement parameters (Å^2^)

**Table:**

	*U*^11^	*U*^22^	*U*^33^	*U*^12^	*U*^13^	*U*^23^
Br1	0.03162 (14)	0.0648 (2)	0.05259 (17)	0.01473 (19)	−0.00240 (17)	0.0052 (2)
O1	0.0350 (15)	0.0224 (14)	0.0629 (18)	0.000	−0.0084 (15)	0.000
C1	0.0301 (13)	0.0443 (16)	0.0257 (13)	0.0091 (16)	0.0001 (11)	0.0004 (15)
C6	0.0487 (17)	0.0390 (16)	0.0299 (13)	0.0231 (19)	−0.0067 (15)	−0.0083 (12)
C3	0.0245 (13)	0.0250 (13)	0.0260 (12)	0.0011 (12)	0.0036 (13)	0.0030 (12)
C7	0.0270 (13)	0.0304 (15)	0.0452 (16)	−0.0031 (13)	0.0024 (12)	−0.0083 (12)
C4	0.0331 (13)	0.0259 (13)	0.0230 (11)	0.0045 (14)	−0.0011 (12)	0.0022 (12)
C2	0.0321 (15)	0.0282 (13)	0.0283 (12)	0.0001 (12)	0.0023 (15)	−0.0005 (12)
C5	0.0489 (18)	0.0275 (15)	0.0321 (13)	0.0069 (16)	−0.0108 (14)	−0.0061 (12)

### Geometric parameters (Å, °)

**Table:**

Br1—C1	1.904 (2)	C3—C4	1.410 (3)
O1—C7^i^	1.422 (3)	C3—C7	1.497 (3)
O1—C7	1.422 (3)	C7—H7A	0.9700
C1—C2	1.375 (3)	C7—H7B	0.9700
C1—C6	1.386 (3)	C4—C5	1.383 (3)
C6—C5	1.389 (3)	C4—C4^i^	1.489 (4)
C6—H6A	0.9300	C2—H2A	0.9300
C3—C2	1.388 (3)	C5—H5A	0.9300
			
C7^i^—O1—C7	113.8 (2)	O1—C7—H7B	108.7
C2—C1—C6	121.8 (2)	C3—C7—H7B	108.7
C2—C1—Br1	119.40 (19)	H7A—C7—H7B	107.6
C6—C1—Br1	118.8 (2)	C5—C4—C3	119.3 (2)
C1—C6—C5	118.3 (2)	C5—C4—C4^i^	121.75 (17)
C1—C6—H6A	120.9	C3—C4—C4^i^	118.96 (16)
C5—C6—H6A	120.9	C1—C2—C3	119.8 (2)
C2—C3—C4	119.5 (2)	C1—C2—H2A	120.1
C2—C3—C7	120.5 (2)	C3—C2—H2A	120.1
C4—C3—C7	120.0 (2)	C4—C5—C6	121.4 (3)
O1—C7—C3	114.29 (19)	C4—C5—H5A	119.3
O1—C7—H7A	108.7	C6—C5—H5A	119.3
C3—C7—H7A	108.7		

Symmetry codes: (i) −*x*+2, *y*, −*z*+1/2.
